# The role of cellular senescence in ovarian aging

**DOI:** 10.1038/s41514-024-00157-1

**Published:** 2024-07-20

**Authors:** Jéssica D. Hense, José V. V. Isola, Driele N. Garcia, Larissa S. Magalhães, Michal M. Masternak, Michael B. Stout, Augusto Schneider

**Affiliations:** 1 Aging & Metabolism Research Program, Oklahoma Medical Research Foundation, Oklahoma City, OK USA; 2 Nutrition College, Universidade Federal de Pelotas, Pelotas, RS Brazil; 3 Burnett School of Biomedical Sciences, University of Central Florida, Orlando, FL USA; 4 Oklahoma City Veterans Affairs Medical Center, Oklahoma City, OK USA

**Keywords:** Senescence, Ageing

## Abstract

This review explores the relationship between ovarian aging and senescent cell accumulation, as well as the efficacy of senolytics to improve reproductive longevity. Reproductive longevity is determined by the age-associated decline in ovarian reserve, resulting in reduced fertility and eventually menopause. Cellular senescence is a state of permanent cell cycle arrest and resistance to apoptosis. Senescent cells accumulate in several tissues with advancing age, thereby promoting chronic inflammation and age-related diseases. Ovaries also appear to accumulate senescent cells with age, which might contribute to aging of the reproductive system and whole organism through SASP production. Importantly, senolytic drugs can eliminate senescent cells and may present a potential intervention to mitigate ovarian aging. Herein, we review the current literature related to the efficacy of senolytic drugs for extending the reproductive window in mice.

## Ovarian reserve and reproductive lifespan

The ovarian reserve is predominantly represented by primordial follicles, a structure composed of an oocyte surrounded by a single layer of pre-granulosa cells, which remains in a quiescent state^1^. When activated, primordial follicles initiate an irreversible process of development, which can lead to ovulation or atresia^2^. The activation of primordial follicles is regulated by several growth pathways. Activation of mammalian target of rapamycin (mTOR) in the pre-granulosa cells of the primordial follicle increases the expression of Kit ligand (KITL), which binds to its tyrosine kinase receptor KIT in the oocyte. This activates the phosphoinositide 3-kinase (PI3K)/protein kinase B(AKT) pathway in the oocyte, culminating in the phosphorylation of forkhead box O3a (FOXO3a) in the nucleus and the activation of the primordial follicle^3^. The progression of ovarian aging is characterized by a gradual reduction, both in quantity and quality, of the oocytes in the ovarian cortex^4^. Accumulated damage with age in oocytes results in embryonic malformation and an increased rate of miscarriage^5^. Therefore, ovarian aging is identified as the primary cause of infertility in females^6^. This decline in the number of primordial follicles is observed with the advancement of age in women^4^ and in mice^7^. A reduction in fertility is observed around the age of 35 years in women, followed by menstrual irregularities around 45 years of age^4^. Infertility is observed in the subsequent years, and eventually, the onset of menopause, characterized by the end of menstrual cycles around 50 years of age^4^. In C57BL/6 mice, the ovarian reserve is reduced by half by 10 months of age, and by 18 months, it is reduced by approximately 10 times, compared to young adult mice^7^. Cyclicity is also interrupted between 11 and 16 months of age^8^, resembling the timeline observed in women. Furthermore, female C57BL/6 mice will cease producing offspring around 18 months of age, with a significant reduction in fertility already observed at 10 months of age^7,9^. This indicates that even though menopause is not a phenomena observed in mice, there is a severe reduction in ovarian reserve and a decrease in ovulation and fertility, similar to what occurs in women^4^. This phenomena has been recently referred as oopause, indicating the end of reproductive cyclicity in mammalian species^10^. This evidence also suggests mice as a valuable model to study reproductive aging.

The cyclical production of female reproductive hormones plays a significant role not only in follicular development but also in women’s overall health^11^. Antral follicles are the primary source of ovarian estrogen secretion in women of reproductive age^12^. Due to the reduction in ovarian reserve, the number of antral follicles also reduces with age, decreasing ovarian steroid hormone output, and affecting female health^13^. As the ovulatory cycles end due to severe depletion of ovarian reserve with age, there is a drastic drop in circulating levels of estradiol, marking the onset of menopause^14^. Thus, menopause is associated with increased metabolic dysfunction and mortality^4,15^. During menopause, women experience a variety of symptoms and conditions associated with changes in steroid levels and aging^16^. This stage of reproductive life significantly increases susceptibility to various metabolic diseases, including cardiovascular diseases^17^, osteoporosis^18^, hypertension, diabetes mellitus^19^ and ovarian cancer^16^. Women experiencing early menopause (<45 years) further increase these risks, with a substantial impact on public health^4,19^. In mice, a similar process is observed. Female mice tend to live longer than males, however, when ovaries are surgically removed, life expectancy is reduced^20^, indicating a clear relationship between reproductive and somatic longevity. Indeed, old female mice receiving ovarian transplants from young mice experience an increase in longevity^21^.

The reduction of estradiol levels is one of the primary effects of menopause, and several studies highlight the benefits of exogenous estradiol replacement therapy^22^. However, longevity extension from ovarian tissue transplantation was higher when young ovaries were depleted of follicles with VCD (4-vinylcyclohexene diepoxide) before transplantation^23^. Additionally, women with more reproductive cycles are at an increased risk of certain pathologies^24^. In mice, cycling generates a microenvironment that may exacerbate the effect of estrogen-dependent mutagenesis in the endometrium^25^. Therefore, while preserving the ovarian reserve is beneficial for lifespan extension in most studies in mice and humans, it may contribute to the development of certain pathologies due to increased number of cycles tissues are exposed.

## The role of senescent cells in the ovary

The aging process differs from tissue to tissue^26,27^. The primary feature of aging in most tissues is the accumulation of senescent cells^28^. Cellular senescence is a state of permanent cell cycle arrest triggered in response to numerous stressors, aiming to inhibit the proliferation of aged and/or damaged cells^29^. Despite this, senescent cells are metabolically active and secrete inflammatory cytokines, chemokines, growth factors, and matrix metalloproteinases^30^. These factors are commonly referred to as the senescence-associated secretory phenotype (SASP)^31,32^. The SASP allows senescent cells to modulate pathways in neighboring and distant cells and tissues^33^ and has been widely used as a marker of cellular senescence^34,35^. The SASP recruits immune cells, thereby creating a pro-inflammatory microenvironment in injured or aging tissues^29,36^. It is important to emphasize that senescence plays physiological roles during normal development and is essential for tissue homeostasis^37^. However, the chronic accumulation of senescent cells with advancing age results in detrimental effects on health, increasing age-related diseases^38,39^. Interestingly, the injection of senescent cells into young mice promotes dysfunctions similar to those observed during chronological aging^40^. This indicates that senescent cells play an active role in the aging process of the organism.

The age-related increase in senescent cells is associated with numerous age-related diseases^41^ including diabetes mellitus^42^, atherosclerosis^38^, Alzheimer’s/Parkinson’s^43^ and inflammatory diseases^44^. However, mice experiments revealed that the number of senescent cells can vary between tissues in the same individual^45^. For the identification of senescent cells, a commonly used strategy is to measure proteins and transcripts of senescence effectors, including p16INK4a (*Cdkn2a*), p53, and p21 (*Cdkn1a*)^46^. Evidence suggests that p53-21 signaling initiates, while p16INK4a signaling is necessary for the maintenance of the senescence state^47,48^. Additionally, increased senescent cell numbers are associated with the accumulation of senescence-associated β-galactosidase (SA-β-Gal)^49^ and lipofuscin^50^. However, senescent cell distribution in tissues is heterogeneous and sometimes hard to determine. In this sense, innovative methodologies, such as spatial transcriptomics, spatial epigenomics, spatial metabolomics can be applied to detect senescent cell activity in each tissue of interest^51^. Genetic editing of human pluripotent stem cell (hPSCs) was also used to generate senescent cell models^52^. Others proposed a method called image flow cytometry, which is based on the analysis of cell autofluorescence and morphological parameters with the use of artificial intelligence and machine learning. This method has proven to be simple and fast and can be used regardless of the type of senescent cells^53^. Although these evidence suggest that senescence markers increase with age in mice, many undergo significant changes only after 12 months of age^34,54^. As mentioned before, mouse fertility is already compromised at 10 months of age, with a severe reduction in ovarian reserve. Therefore, it is necessary to better understand the role of cellular senescence in the ovary, as this organ shows impairment of its functions much earlier than other tissues.

There is limited data on senescence cell accumulation and their function in the ovary. Although there is no well-defined panel of biomarkers for cellular senescence, some have been widely used in the ovary, including markers of pro-inflammatory stress, double-strand DNA breaks, and lipofuscin^55–57^. Corresponding with reduced ovarian function, there is also a significant increase in markers related to senescence in the ovaries of mice between 3 and 12 months of age (*Cdkn1a*, *Cdkn2a*, *Pai*-1, and *Hmgb1*), along with the accumulation of lipofuscin aggregates^58^. Similar accumulation of senescent cells in other organs is observed much later in life, around 18-20 months of age. Additionally, the ovarian transcriptomic profile indicates a positive regulation of genes related to pro-inflammatory stress and cell cycle inhibition, while genes involved in cell cycle progression were negatively regulated, which is characteristic of senescent cells^58^. Increased SA-β-Gal and p21 levels was detected in the ovarian stroma of mice at 8-10 months of age, indicating senescent cell accumulation^59^. An increase in the burden of senescent cells, identified through SASP markers, was also observed when cisplatin was used to induce damage in the ovaries of very young mice^60^. An increase in p16 & p21 expression and lipofuscin aggregates were observed in the ovaries of genetically obese mice compared to lean controls as early as six months of age^61^. This indicates that markers of senescence in ovarian tissue can be observed before 12 months of age in mice. Similar observations were made in human tissue. Expression of p21 was elevated in ovarian of middle-aged women (>37 years) compared to young controls (<33 years)^62^. Other senescence and fibrosis related genes were also up-regulated in stromal cells of middle aged compared to younger women^62^. Expression of these senescence biomarkers are often exacerbated when stressors such as chemotherapy treatment and obesity are present.

As the ovary ages, there is also a well-defined increase in inflammation and changes to extracellular matrix (ECM), including increased collagen deposition^63,64^. Senescent cells produce a SASP signature, which is often used to identify its tissue accumulation, as discussed earlier. SASP components include soluble factors, such as pro-inflammatory cytokines and proteases involved in tissue remodeling (matrix metalloproteinases [MMPs] and tissue inhibitor of metalloproteinases [TIMP]), but also includes insoluble factors that are ECM components, such as collagen and lamin^65^. Therefore, it is challenging to separate senescent cells accumulation from the physiological ovarian response of tissue remodeling and accumulation of inflammatory cells observed with age. Pro-inflammatory responses play a significant role in ovarian aging^55,66^. Most age-related transcriptional changes observed in the ovaries of mice are associated with inflammation and immune responses^67^. Recent proteomic evaluations appear to mirror these findings^68^. Age-related increases in cytokines such as interleukin (IL)-6, IL-1β, IL-8, interferon-γ (Infg), TNF-α, and chemokines, such as, monocyte chemoattractant protein-1 (Ccl2), CC motif chemokine ligand (Ccl) 5, and CXC motif chemokine ligand (Cxcl) 2 have been reported in the ovaries of mice^55,64,69^. Studies in humans have revealed similar findings, with elevated levels of IL-3, IL-6, IL-7, IL-15, Ccl3, and Cxcl10 in the follicular fluid of older women^70,71^. A recent single-cell transcriptomic and flow cytometry analysis showed age-related accumulation of immune cells in the ovary^72^. The same report also showed that senescence-associated genes in the ovary are predominantly expressed by immune cells and generally did not change with age. Hence, increased in cellular senescence during ovarian aging may reflect increased immune cell abundance. Interestingly, *Cdkn1a* expression increased in Type 17T lymphocytes with age, suggesting that this cell population might enter a senescence state in the ovary. Aged ovaries also accumulate multinucleated giant cells (MNGCs), which are believed to result from the fusion of macrophages associated with chronic inflammation^55,73^. Lipofuscin positivity is considered a senescence marker, however it colocalizes with MNGC, suggesting that either lipofuscin is staining MNGCs, or MNGCs are also in a senescent state^72^.

Age-related pro-inflammatory stress in the ovary is a conserved phenotype across species and may be causally linked to the decline in oocyte quality, challenges in conception, and the onset of declining fertility^4^. Dysregulation of immune cells and/or altered inflammatory signaling has been implicated in reproductive declines^74^. It is known that pro-inflammatory mediators increase the activation of primordial follicles, causing damage in oocyte/embryonic development and decreasing the production of steroid hormones^75^. Conversely, IL-1 knockout mice have increased ovarian reserve and fertility^57^. Although the expression of collagen synthesis genes remains constant in the ovary during the reproductive window^69,72^, there appears to be a decrease in the activity of collagenase^72^, resulting in the accumulation of collagen and increased tissue stiffness. In turn, this stiffness can hinder ovulation^64^, and lead to a reduction in fertility in older mice. Nevertheless, macrophages play critical roles in multiple aspects of ovarian functions. Macrophages can be activated and polarized to the M1 or M2 subtype depending on the pro-inflammatory or anti-inflammatory stimulus, respectively^76^. The increase in M1 macrophages has been associated with increased primordial follicular activation, while the increase in the M2 subtype had inhibitory effects^76^. The ovulatory process itself is preceded by inflammatory reactions that occur in mature follicles^77^. Single-cell analysis of ovary, oviduct, uterus, cervix, and vagina indicate these organs undergo recurrent immune infiltration and ECM remodeling in each cycle^25^. In the uterus, oviduct, and vagina, fibrotic tissue accumulated with aging and continuous cycling. Mice that were induced to estropause had reduced fibrotic tissue in uterus and oviduct, due to reduced number of ovarian cycles^25^. Ovarian fibrosis seems to be aggravated by persistent chronic inflammation^62^. This in turn can be due accumulation of pyroptotic macrophages which interact with effector T cells and secrete factors promoting pyropoptosis in a feedback loop^62^. Therefore, inflammation and tissue remodeling are present in the reproductive tract at all stages of aging, making it difficult to identify the physiological and pathological roles of senescence in this context. However, it also points to modulation of the SASP as an important step in improving fertility.

## The use of senolytics to curtail female reproductive aging

Senolytics drugs eliminate senescent cells and have been reported to increase longevity in mice^40^. Several compounds have been studied for their senolytic properties, including quercetin, dasatinib, fisetin, resveratrol, curcumin, and others^78^. Quercetin was reported to have senolytic potential in combination with dasatinib^79^. Quercetin is a flavonoid found in fruits and vegetables that has antioxidant^80^, anti-inflammatory, and antineoplastic properties^81^. It inhibits the PI3K signaling pathway and indirectly stimulates the apoptosis of senescent cells^82^. Dasatinib is a multi-tyrosine kinase inhibitor used in cancer treatment^83^. Dasatinib targets the ephrin B survival-regulating dependency receptor (Efbn1)^82^ and anti-apoptotic pathways activated in senescent cells, such as Bcl-2 and Bcl-xL^78^. Fisetin is also a member of the flavonoid family, found in low concentrations in fruits and vegetables^84^. It has antineoplastic, neuroprotective, and antioxidant effects^84^ and has been widely studied for its senolytic effect^85^. Fisetin also targets the anti-apoptotic pathways Bcl-2 and Bcl-xL^86^ and inhibits Pi3k/Akt and mTOR pathways^87^.

The combined use of dasatinib and quercetin (D + Q) selectively targets a broad range of senescent cell types more effectively than when used individually^79^. D + Q treatment was shown to be safe both in vitro and in vivo, causing the selective elimination of senescent cells and promoting increased lifespan when administered to old mice (19–21 months old)^40^. Studies have also shown success with the use of the D + Q senolytic cocktail in improving symptoms of age-related diseases in humans with idiopathic pulmonary fibrosis^88^ and Alzheimer’s disease^89^. Fisetin also has a senolytic effect, clearing senescent cells and reducing SASP-induced inflammation in vitro and in vivo^90^. Only one month of fisetin treatment increased the lifespan of progeroid and old wild-type mice (22–24 months of age)^85^. Some studies also tested the efficacy of senolytics in disease models, such as lupus, atherosclerosis, obesity and Alzheimer^61,91–96^. However, most of these studies were performed with old (19–24 months old) or progeroid mice^40,85^. Therefore, few studies target healthy females within the reproductive window, typically up to approximately 15 months of age. This implies that senolytics are used when the ovarian reserve is already depleted. As the primordial follicle reserve is not renewed, the damage to fertility is irreversible at this point. Additionally, it is not clear from current studies if the amount of senescent cells accumulated in the ovaries during this reproductive age is sufficient to have any impact in fertility when cleared by senolytic treatments.

In this regard, a reduction in the levels of p21, p16, and lipofuscin was observed in the ovaries of genetically obese mice treated with D + Q at six months of age^61^. However, we observed that D + Q treatment in healthy females from one six months of age did not affect ovarian reserve or fertility^97^. Additionally, treatment of females with D + Q or fisetin from six to 12 months of age also did not affect ovarian reserve and fertility^97^. Fisetin reduced macrophage infiltration and lipofuscin staining in ovarian tissue^97^. Some SASP markers were reduced while others were increased by senolytic treatment^97^. This indicates a small effect of senolytics in ovarian senescence markers, which did not translate into preserved ovarian reserve or improved fertility. Granulosa cells treated with quercetin alone had attenuated cell injury and aging induced by H_2_O_2_, activating protective autophagy and up-regulating autophagy-related proteins^98^. This suggests that quercetin alone could improve ovarian function. Treatment of young female and male mice (4–13 months old) with D + Q and fisetin has controversial effects. While fisetin improved glucose and energy metabolism, reduced SASP, and enhanced cognitive performance in young male mice, D + Q had minimal effects^99^. Conversely, D + Q was detrimental in females, increasing SASP expression and fat accumulation, reducing energy metabolism, and cognitive performance, while fisetin had no significant effects^99^. These findings indicate that age and sex are key determinants of the effectiveness of senolytics. Taken together, these data suggest that the use of senolytics in young female mice of reproductive age does not benefit ovarian reserve and fertility and may even have detrimental effects.

In this context, it is known that chemotherapy agents contribute to infertility, early menopause, and premature ovarian failure in young women undergoing cancer treatment^100^. Chemoterapy agents cause an hyperactivation of primordial follicles through massive apoptosis of growing follicles and reduced AMH levels^101^. Young female mice (6 weeks old) received cisplatin one week after starting a four-week course of D + Q treatment. Cisplatin reduced the ovarian reserve and induced ovarian senescence, as demonstrated by in vitro staining of granulosa cells with SA-β-Gal and in vivo immunohistochemistry for SA-β-Gal, p16, p21, and IL-6^60^. Treatment with D + Q reduced the senescent cell burden and preserved the ovarian reserve, reducing DNA damage and ovarian fibrosis, resulting in increased litter sizes^60^. Similarly, mice treated with the chemotherapeutic agent doxorubicin had increased ovarian senescent cell burden. The accumulation of senescent cells in the ovaries after doxorubicin treatment was determined through increased SA-β-Gal, p16 and p21^102^. Once again, D + Q and fisetin reduced senescent cell burden in treated mice. However, senolytics did not reverse the follicular loss and ovarian stromal fibrosis caused by doxorubicin^102^. In this experiment, the dose of doxorubicin was administered on the day following the first administration of senolytics D + Q, and the treatment lasted for three weeks. Conversely, when mice received the chemotherapeutic one week after starting treatment with senolytics, positive effects in the ovarian reserve were observed^60^. This suggests that senolytics may be acting to prevent damage caused by chemotherapeutical agents rather than being able to reverse any damage caused by established senescent cells and subsequent depletion of the ovarian reserve.

Nicotinamide adenine dinucleotide (NAD) also emerges as a promising regulator in attenuating age-related functional decline and associated diseases. NAD metabolism can influence SASP production by senescent cells^103^. As ovarian aging progresses, there is a reduction in NAD levels, which presents promising strategies for intervention^104,105^. Disruption of the NAD pathway results in decreased ovarian NAD levels, mitochondrial dysfunction, diminished ovarian reserve, and reduced oocyte quality in old mice^106^. This highlights the potential of NAD precursors supplementation to preserve oocyte quality and ovarian health. CD38, one of the enzymes that consumes NAD^107^, is predominantly expressed in the ovarian extrafollicular space, especially in immune cells, and its levels increase with age^108^. The absence of CD38 results in increased ovarian NAD levels and fertility in young mice, associated to a greater initial ovarian reserve^108^. Given the link between NAD levels and SASP production, more studies focused on ovarian senescent cells are needed to understand its role in fertility.

## Future directions

There are few studies using senolytics in young reproductive age mice available in the literature, which suggest that the compounds currently used have few beneficial systemic benefits at this age window. Even fewer studies evaluated the effects of senolytics in the ovary. These suggest that senolytics may prevent ovarian reserve loss, but cannot reverse the damage to the ovarian reserve after senescence is established. The activation of primordial follicles is an irreversible process, which means that the damage promoted by senescent cells in the ovarian reserve would not be able to be reverted by senolytics. This may indicate the direction for future studies, focusing on preventing accumulation of senescent cells in the ovaries in order to prevent declines in fertility. Additionally, the inflammation generated by senescent cells through the SASP itself can contribute to irreversible follicular activation. Therefore, it is possible that other senolytic compounds with greater efficacy in the ovary need to be tested. Compounds with senomorphic activity, i.e. able to decrease SASP secretion, may be considered to prevent the negative pro-inflammatory environment generated by senescent cells in the ovary. Furthermore, the physiological role of senescent cells in reproductive functions must be considered, as inflammation and tissue remodeling are key points in the ovulatory process in females. Therefore, the path to validating the use of senotherapies in female reproductive aging is still open. A better understanding of ovarian senescence biomarkers and the role of senescent cells on female fertility is still necessary in order to define how to promote targeted elimination of these cells without negative impact on other organs in young females.
